# The Effect of Contract-Rearing on the Health Status of Replacement Dairy Heifers

**DOI:** 10.3390/ani11123447

**Published:** 2021-12-03

**Authors:** Marie-Claire McCarthy, Luke O’Grady, Conor G. McAloon, John F. Mee

**Affiliations:** 1 Dairy Production Research Centre, Teagasc, Animal and Bioscience Research Department, Moorepark, Fermoy, P61 P302 Co. Cork, Ireland; marieclairemaccarthy@gmail.com; 2 School of Veterinary Medicine, University College Dublin, Belfield, D04 V1W8 Dublin, Ireland; luke.ogrady@ucd.ie (L.O.); conor.mcaloon@ucd.ie (C.G.M.); 3 School of Veterinary Medicine and Science, University of Nottingham, Nottingham LE12 5RD, UK

**Keywords:** contract-rearing, dairy heifers, health status

## Abstract

**Simple Summary:**

In this study, we set out to compare the health of contract- vs. conventionally reared replacement dairy heifers. Contract-reared heifers are raised off-farm on another farm by another farmer, for a fee. A total of 120 dairy farmers were enrolled in the study: 55 farmers were rearing their own heifers (control farmers; CFs), and 65 were sending heifers to a contract-rearing farm (source dairy farmers; SDFs). Over two years, we monitored approximately 5500 replacement heifers from these farms to check for signs of ill health using a calf health scoring system that involved individually examining each animal at four farm visits (twice annually). Additionally, faecal and nasal swabs were taken from a proportion of heifers with clinical signs of diarrhoea and respiratory disease. Overall, the results indicated few differences in the health and infectious status of home-reared versus contract-reared heifers. Additionally, the number of source dairy farms represented and mixing of heifers from multiple farms at the rearing unit were not associated with an increased incidence of respiratory disease or diarrhoea among contract-reared heifers. Therefore, it was concluded that contract-rearing did not result in adverse health outcomes for replacement dairy heifers.

**Abstract:**

The aim of this study was to compare the health status of contract- vs. conventionally reared replacement dairy heifers over a 2-year period. A total of 120 dairy farmers were enrolled in the study in spring 2018: 55 farmers were rearing their own heifers (control farmers; CFs), and 65 were sending heifers to a contract-rearing farm (source dairy farmers; SDFs). Between spring 2018 and autumn 2019, approximately 5500 replacement heifers from these farms were monitored for signs of ill health during four farm visits using a modified version of the Wisconsin calf health scoring system. Additionally, faecal and nasal swabs were taken from a proportion of heifers with clinical signs of diarrhoea and respiratory disease to determine the associated aetiological agents. Results indicate few differences in the health status and pathogen exposure status of home-reared versus contract-reared heifers. Additionally, the number of source dairy farms represented and commingling of heifers from multiple origins at the rearing unit were not associated with an increased incidence of respiratory disease or diarrhoea among contract-reared heifers. It was concluded that contract-rearing did not result in adverse health outcomes for replacement dairy heifers. This is the first study to demonstrate this finding in a robust, longitudinal, herd-level population study.

## 1. Introduction

Contract-rearing of replacement heifers is proposed as a solution to overcome the challenges associated with land and labour shortages experienced by dairy farmers, particularly those who wish to expand their herd following the abolition of EU milk production quotas in 2015. Typically, heifers are moved from their source herd to the rearing unit before or at weaning and return at the point of calving [1]. Although contract-rearing may serve as a tool to assist farmers in achieving their herd expansion goals, inherent disease transmission risk factors associated with the practice may limit its future uptake. Between-herd movement of cattle represents a major transmission route for several infectious diseases [2], and because contract-rearing involves the movement of animals between their source herd and one or more rearing units which, in turn, may be raising heifers for multiple dairy herds, the potential risk of inter-herd disease transmission is high.

Potential challenges to heifer health associated with contract-rearing include the commingling of cattle of unknown disease status from multiple sources, an important risk factor for several infectious diseases, particularly bovine respiratory disease (BRD) [3,4]. Commingling of cattle from various sources is also likely to illicit a stress response due to the disruption of social structures, compounding the immunosuppressive effects of recent weaning, transportation and adaption to a new environment, which are intrinsic features of contract heifer rearing [5]. Additionally, suboptimal colostrum feeding practices on dairy farms engaged in contract-rearing [1] can place heifers at risk for failure of the passive transfer of immunoglobulins with resultant increased susceptibility to infection [6].

The economic implications of disease events during the heifer rearing period are twofold: some costs associated with the treatment of sick animals, and potential losses associated with impaired future milk production performance, reproductive capacity, and reduced survival [7,8]. Lifetime effects of calf-hood disease include increased age at first calving, reduced feed efficiency, poor fertility, and premature culling and mortality [7,9,10]. Enteric and respiratory diseases are the leading causes of morbidity and mortality among calves under 6 months old on Irish dairy farms, followed by umbilical infection [11]. Previous studies have estimated the incidence of diarrhoea and respiratory and navel infections among conventionally reared calves under 6 months old on Irish dairy farms at 24%, 3.5% and 1.4%, respectively [12]. The morbidity of contract-reared heifer calves in Irish pasture-based systems of milk production has not previously been described. As a result, the aim of this study was to characterise and compare the health outcomes of conventionally and contract-reared dairy heifers over a 2-year period.

## 2. Materials and Methods

### 2.1. Ethical Authorization

This study was conducted between February 2018 and December 2019. Ethical approval to conduct the study was obtained from the Teagasc Animal Ethics Committee (TAEC177-2017), and procedure authorization (AE19132/P075) was granted by the Health Products Regulatory Authority of Ireland (HPRA). Experiments were undertaken in accordance with the European Union (Protection of Animals Used for Scientific Purposes) Regulations 2012 (S.I. No. 543 of 2012).

### 2.2. Study Population

Herds were recruited to this study in spring 2018, as previously described in McCarthy et al., 2021 [1]. Briefly, through a multistep process involving the national cattle breeding database (ICBF) and several Irish farming stakeholder bodies and media awareness campaigns, one hundred and twenty dairy farmers were recruited into a 3-year, nationwide, longitudinal study to investigate the risks to animal health associated with contract heifer rearing.

Farmers sending heifers to be reared at a contract-rearing facility were classified as source dairy farmers (SDFs), and farmers rearing their own heifers were classified as control farmers (CFs). From the respondent pool, source dairy farmers were randomly selected for inclusion. Control dairy farmers were subsequently selected based on their geographical proximity to the recruited source dairy farms. In total, 120 herds were recruited to the study in spring 2018: 65 were source dairy herds and 55 were control herds. Concurrently, the contract-rearing (CR) farms taking heifers from recruited source dairy herds were also recruited into the study (*n* = 57). Thus, a single cohort of spring-born calves was followed longitudinally throughout the study.

The majority of the recruited dairy herds were spring calving (92%), whereas the remainder were operating under a split spring–autumn calving system. All regions within Ireland were represented; however, the highest density of farms was in county Cork, reflecting the distribution of the national dairy cow population. Participation in the study was voluntary and non-incentivized. To establish the number of source and control farms (the observational unit) required to detect a clinically relevant difference in the age at first calving (AFC) of 30 days between groups, a power calculation was performed. Using a two-tailed test at 5% confidence level with a power of 80%, 53 herds/group were required. This metric, AFC, was chosen as the single critical key performance indicator (KPI) of all aspects of heifer-rearing management, including nutrition and health, particularly in a seasonal calving management system, such that predominates in Ireland. To allow for herd drop-out over the course of the three-year study, extra herds were recruited where possible

### 2.3. Farm Visits

Between spring 2018 and autumn of 2019, study farms were visited on four occasions. During the first farm visit period between February and April 2018, heifers were approximately 1 month old (median 39 days, range: 9–93). All initial farm visits were conducted on the heifers’ farm of origin. For conventionally reared heifers, subsequent visits (visits 2, 3 and 4) were also conducted on the farm of origin. For contract-reared heifers, however, all subsequent farm visits were conducted at the rearing unit. The second farm visit was conducted between September and December 2018, when heifers were approximately 8 months of age (median; 261 days, range; 139–330). The third and fourth farm visits took place when heifers were approximately 12 months old (median; 386 days, range; 265–471) in spring 2019, and 20 months old (median 612 days, range; 481–694) in autumn 2019. Approximately 6500 heifers were enrolled in the study at the first farm visit. Over the course of the study, loss of heifers occurred due to farm drop-out (*n* = 7), mortality and the sale of heifers, resulting in data being available for 5532 heifers for all four data collection time-points.

### 2.4. Health Scoring

At each farm visit, female calves born in the spring of 2018 were identified and their unique official 12-digit ear tag number was recorded. Each heifer was clinically assessed and a health score of 0 to 3 [normal (0), very abnormal (3)] was assigned to each clinical parameter using a modified version of the Wisconsin clinical health scoring system [13]. Scores were recorded for each of the following clinical parameters: ocular discharge, nasal discharge, rectal temperature, presence of cough, navel abnormalities, appearance of joints and faecal consistency (Table 1) (available at https://www.vetmed.wisc.edu/fapm/svm-dairy-apps/calf-health-scorer-chs/, accessed on 2 December 2021). The scoring system was modified to exclude the assessment of ear position due to the possibility of confounding associated with injurious ear tag placement. Faecal consistency was assessed by the observation of faecal material on the perineal and tail regions of calves, and by the examination of faeces produced during the time in which the calf was being examined (for example, while recording rectal temperature). Health scores were recorded by two trained observers. After the initial farm visit, the scoring of navel and joint characteristics were discontinued due to safety issues associated with navel palpation in older animals in crush/race/chute handling facilities.

Notably, because bovine viral diarrhoea (BVD) was being mandatorily eradicated from Irish cattle herds while this study was conducted, sampling for BVDv was not conducted by the research team; all newborn calves nationally were ear-tissue-tag biopsy-sampled after birth and tested for BVDv by PCR in a government-approved veterinary diagnostic laboratory.

### 2.5. Upper Respiratory Tract Sampling

At the initial farm visit, nasal swabs were collected from untreated heifers with clinical signs of respiratory disease (score of ≥2 for nasal discharge and/or cough) (up to a maximum of 4 calves/farm). For virology, a nasal mucous sample was obtained by inserting a sterile plain cotton swab (Sarstedt AG, Nümbrecht, Germany) into the ventral meatus to a length of approximately 12 cm. Care was taken to avoid the swab touching the nares. The swab was rotated 360° several times against the mucous membrane, withdrawn, and the procedure repeated with the same swab on the contralateral side. For bacteriology, this procedure was repeated, and the swab (Sarstedt AG, Nümbrecht, Germany) was placed in Amies agar gel. Samples were labelled on-farm and placed in a cooler box until postage to the laboratory upon return to the research centre. In total, 81 samples from 22 SDFs and 17 CFs were submitted for analysis. Nasal swabs were individually tested at the central veterinary research laboratory (CVRL) in Backweston, Ireland, for the bacterial and viral agents outlined in Table 2 using the methods described by O’Neill, Mooney [14].

### 2.6. Faecal Sampling

Faecal samples were taken from up to 5 heifers per farm with clinical signs of diarrhoea and pyrexia at the first farm visit in spring 2018 (faecal consistency score of ≥2; tail visibly stained; and temperature > 39.5 °C). Samples were taken from the rectum of calves using plain cotton swabs (Sarstedt AG, Nümbrecht, Germany) and stored in a cooler box until return to the research centre. Samples were analysed on the day of collection with a commercially available chromatographic lateral flow immunoassay (Zoetis WITNESS^®^ BOVID-5 diagnostic test kit). These kits were capable of identifying the following pathogens in bovine faecal samples: *E. coli* K99, *Giardia lamblia, Cryptosporidium* spp., coronavirus, and rotavirus. Where a positive result was obtained for cryptosporidium spp., it was assumed to be *Cryptosporidium parvum*, the most common species found to cause diarrhoea in young calves [15]. In total, 221 samples from 47 SDFs and 31 CFs were collected and tested. The reported sensitivity and specificity of the diagnostic test kit is outlined in Table 3 [16]. Faecal samples were also submitted to the CVRL in Backweston for bacteriological culture analysis to detect the presence of *Salmonella* Dublin.

### 2.7. Data Analysis

Analyses were conducted using SPSS 24.0 (SPSS Inc., Chicago, IL, USA). At calf-level, chi-squared procedures were used to report the distribution of health scoring variables on each farm type (SDF or CF), including temperature, nasal discharge, ocular discharge, faecal consistency, and joint and navel abnormalities. Chi-squared procedures were also used to detect differences in the distribution of respiratory and enteric pathogens identified in nasal and faecal swabs taken from heifers on source and control dairy farms during the initial visit period.

Health scores were dichotomised using clinical thresholds. Rectal temperatures of ≥39.5 °C were considered to be abnormal, in agreement with pyrexia thresholds in similar studies [17]. For the remaining health outcomes, a score of ≥1 was considered abnormal. The cumulative scores of rectal temperature, nasal and eye discharge, and cough were used to calculate an overall respiratory score. Calves with a score of ≥5 (out of a possible 15) were considered to have bovine respiratory disease [13]. Calves with a faecal score of ≥2 were considered to have diarrhoea [18,19]. Calves with a navel score of ≥1 were diagnosed with navel ill.

Descriptive analyses were conducted. For the mean herd-level prevalence of abnormal scores on each farm type, results of heifer-level data were aggregated to herd level to calculate the prevalence of abnormal health scores among heifers within herds (calculated as the number of heifers with a positive outcome divided by the total number of heifers scored). Associations between rearing systems and outcomes at each farm visit period were compared statistically using general linear models. Significant associations were defined at *p* < 0.05.

## 3. Results

### 3.1. Herd Characteristics

Farmers who sent their heifers for contract-rearing (CR) had an average herd size of 198 cows (range 60–380) and sent an average of 64 heifers to the rearing unit. Control farmers had an average herd size of 146 cows (range 60–501) and reared an average of 47 heifers. The most common CR arrangement was one SDF sending heifers to one rearing unit (70%), followed by one SDF sending heifers to multi-origin rearing units (28% of SDFs). Least commonly, source dairy farmers sent heifers to more than one rearing unit (2%). Heifers most frequently arrived at the rearing unit at between 2 and 4 months of age (53% of SDFs) and returned to the SDF between 18 and 21 months of age (56% of SDFs). The associations between herd size (across herd types) and within-herd prevalence of heifer health outcomes during the initial spring farm visit are shown in Table 4.

### 3.2. Health Scoring

#### 3.2.1. Calf-Level

The distribution frequencies of calf-level health scores recorded during the four farm visit periods are shown in Table 5. For each period, and across farm types, the majority of calves were assigned a score of 0 for each health characteristic, i.e., they were healthy. Abnormal health scores for all parameters were most common during the first farm visit period when the median age of heifers was 39 days. For the three remaining visit periods, the number of calves exhibiting signs of ill health declined across farm types, with the exception of nasal scores and pyrexia during the second sampling period.

During the first farm visit period, conducted on the heifers’ dairy farm of origin, a greater percentage of calves on source dairy farms had abnormal nasal discharge, eye discharge and joint scores than on control dairy farms (*p* = 0.001, 0.042 and 0.03, respectively). For the remaining health score outcomes, no differences were evident between farm types. During the second farm visit period (conducted on control dairy farms and contract-rearing farms), a greater proportion of heifers on control farms had pyrexia than heifers on contract-rearing farms (*p* = 0.001). However, a greater percentage of heifers on rearing units had abnormal faecal and nasal scores compared to heifers on control farms (*p* = 0.003 and *p* = 0.001, respectively). During the third visit period (spring 2019), a greater proportion of heifers on control farms experienced pyrexia than contract-reared heifers (*p* = 0.001). For the remaining visit period, when heifers were 12 months and older, no differences were detected in health score outcomes by farm type.

#### 3.2.2. Herd-Level

The overall mean within-herd prevalence of diarrhoea, respiratory disease, and abnormal health score outcomes among heifers on study farms are displayed in Table 6.

The within-herd prevalence of diarrhoea, respiratory disease, and abnormal health score outcomes among heifers on home- and contract-rearing farms are shown in Table 7. During the initial farm visit, before heifers were moved to the rearing unit, a higher prevalence of abnormal cough and nasal scores was recorded among heifers on source dairy farms than those being reared on their farm of origin (*p* = 0.003 and 0.05, respectively). During the second visit period, there was a higher prevalence of pyrexia among home-reared heifers than heifers on rearing units (*p* = 0.04).

### 3.3. Respiratory Tract Sampling

Results from nasal sampling are shown in Table 8. The pathogen most frequently identified across farm types was *Pasteurella multocida*. A greater percentage of samples were positive for *Mycoplasma bovis* on CFs than SDFs (*p* = 0.001).

The majority of samples were positive for at least one respiratory pathogen, irrespective of farm type (Table 9).

### 3.4. Faecal Sampling

*Cryptosporidium parvum* and rotavirus were the pathogens most frequently identified in faecal samples taken from calves with clinical signs of enteritis on both farm types (Table 10).

The majority of faecal samples were negative for all pathogens, and there was no difference in detection rates for multiple enteric pathogens across farm types (Table 11).

### 3.5. Contract-Rearing Arrangements and Heifer Health

No significant associations were observed between the number of farms at the contract-rearing unit and within-herd prevalence of abnormal health scores, diarrhoea and respiratory disease among heifers during the three sampling periods when they were at the rearing unit. The relationship between heifer health outcomes and the number of source dairy farms present (1 or >1 source dairy farms) at the rearing unit is shown in Table 12. The relationship between heifer health outcomes and the practice of commingling heifers at the rearing unit is shown in Table 13.

## 4. Discussion

This was a longitudinal, descriptive study to compare the prevalence of heifer health outcomes on contract- and non-contract-rearing dairy herds. To the best of the authors’ knowledge, this is the first study to investigate the prevalence of diarrhoea, navel ill, and respiratory disease on contract-rearing farms in a pasture-based dairy production system.

Calf-level outcomes for heifer calves on study farms were broadly consistent with the findings of other Irish studies, with most calves having a score of 0 for all parameters, i.e., they were healthy [20,21]. The median age of heifers during the first sampling period was 39 days, just outside the consensual definition of the neonatal period of 30 days [22]. The risk of morbidity was highest for study heifers during this period, consistent with the findings of many morbidity studies conducted in Ireland and internationally [12,23,24]. Diarrhoea was the most common cause of heifer morbidity during this period on both farm types (4% of heifers), followed by respiratory disease (2% of heifers), reflecting the most common causes of mortality in Irish calves of this age [25]. During the second visit period, the median age of heifers was 8.6 months and respiratory disease was the most common cause of morbidity during this period (2% of heifers). Although few studies have reported on the causes of morbidity in heifers of this age, our findings are consistent with post-mortem diagnosis for Irish cattle of this age [25]. No studies have reported on morbidity associated with diarrhoea and respiratory disease in dairy heifers beyond 12 months of age. The epidemiology and risk factors for these infections are almost exclusively associated with animals under 12 months old [26,27]; therefore, the prevalence of these infections was expected to be lowest for heifers during the third and fourth visit periods.

Overall, the herd-level prevalence of diarrhoea, respiratory disease, and navel ill on study farms during the first visit period was lower than those reported in morbidity studies for calves under 6 months old on both dairy farms and specialist calf-rearing units [28,29,30,31,32]. These studies reported cumulative prevalence over multiple farm visits, and as such, are not directly comparable with the current study. Farmers recruited to this study, by their nature, had larger than average, expanding herds, which may have resulted in bias towards more progressive, better-managed herds. This is supported by data on disease prevention measures, which were generally well implemented by study farmers in both cohorts [1], which may have accounted for these lower morbidity estimates.

Although a higher prevalence of abnormal cough and nasal discharge scores was observed among heifers on source dairy farms, when compared to conventionally reared heifers, during the initial spring visit, there was no difference in the prevalence of respiratory disease between farm types, indicating that the severity of these clinical signs was mild. However, the sensitivity of the Wisconsin scoring system has been estimated at 46% for the detection of respiratory disease in a population of sick and healthy calves [33]. As a result, underestimations of BRD diagnoses may have occurred. Additionally, calves exhibiting mild symptoms may be at the preliminary stages of respiratory infection; thus, the relative time of scoring may have influenced the prevalence estimate. Health scores taken during the first sampling period corresponded to baseline data before heifers were moved to the rearing unit, and differences in management practices between control and source dairy farms may have accounted for differences in the prevalence of abnormal nasal and cough scores during this period [1].

Biosecurity and management practices implemented by our study farms have previously been reported [1], and findings of the latter study indicate a greater use of group calving pens by SDFs than CFs, with an average of 18 cows occupying group pens on these farms (versus an average of 12 cows in CF group calving pens). Additionally, individual housing of calves was more common on CFs than SDFs. For farmers using group calf housing, there was a greater tendency among CFs to house calves in groups of 10 or fewer when compared to SDFs. When compared to individual maternity pens, the use of large group maternity pens by SDFs could potentially have resulted in the increased exposure of neonatal calves to respiratory pathogens shed by periparturient cows with subclinical infections. The compact nature of the calving season in pasture-based milk production systems, which predominate in Ireland, results in a large percentage of the herd calving in a relatively short period, with resultant potential for overcrowding in maternity facilities with more cow–calf contact opportunities and for pathogen transmission [4].

Although management practices relating to neonatal calves were similar on both farm types, there was a greater tendency for CFs to feed colostrum to all calves within the first hour of birth as opposed to SDFs. The optimum timing of the first colostrum feed is within 2 h of birth, following which the immunoglobulin absorption ability of the neonatal calf declines [34]. As a result, more rapid colostrum feeding by CFs may have resulted in enhanced immunity of heifers on these farms, conferring them increased protection from respiratory infections [4,35]. The use of larger-group maternity pens on SDFs may further reduce the ability of calves to obtain a sufficient volume of colostrum due to increased chances of cross-suckling and mismothering [36].

Individual calf housing is considered preferable to group housing from a disease prevention perspective, and several studies have reported an association between housing calves in large groups (6–30 calves) in early life and a higher risk of acquiring respiratory infection during the first 210 days of life when compared to calves housed in smaller groups [28,37]. As group size increases, there are more direct contact opportunities between animals and a larger pool of susceptible animals, facilitating spread of infectious agents.

Few further differences in biosecurity and management practices were observed between farm types [1], and few differences in heifer health outcomes were recorded after the initial farm visit, when contract-reared heifers were at rearing farms, indicating comparable heifer health management on rearing units and dairy farms. The herd size of study farms was not found to be associated with herd-level health outcomes during the first visit period, when all heifers were scored on their farm of origin. This is consistent with the findings of an Irish study by Barry et al. [38], who found no association between herd size and calf mortality in the post-quota era of herd expansion.

The bacterial pathogens most frequently identified in nasal samples taken from heifer calves with clinical signs of respiratory disease were *P. multocida, M. haemolytica*, and *M. bovis.* These findings are in agreement with those reported in an Irish post-mortem surveillance study of fatal calf pneumonia cases [25]. The higher prevalence of *M. bovis* in calves on control farms was due to an outbreak of Mycoplasma pneumonia on one study farm.

Viral pathogens were detected in ≤2% and ≤8% of samples submitted from SDFs and CFs, respectively. This detection rate is considerably lower than that reported by O’Neill et al. [14], who detected viral pathogens in 34.6% of nasal swabs taken from Irish dairy and beef calves with clinical signs of respiratory infection under 3 months old. The viral pathogens most frequently identified by O’Neill et al. [14] were bovine coronavirus (22.9%) and BRSV (11.6%). Similarly, bovine coronavirus (5%) and BRSV (5%) were the viral agents most frequently identified in our study, albeit at lower detection rates. Nasal swabs tested in the O’Neill et al. [14] study consisted of both individual and pooled samples taken on farms experiencing respiratory disease outbreaks, which may have accounted for the higher detection rate of viral pathogens when compared to the present study. Additionally, the limited number of samples tested in the present study precludes robust comparison with the O’Neill et al. [14] study outcomes.

Respiratory disease is the most common cause of morbidity and mortality in cattle older than one month; therefore, there are few Irish studies available for direct comparison with our study, because much of the available literature is focused on the agents associated with respiratory disease in cattle between 6 and 12 months of age [39], considerably older than the median age of heifers sampled in the current study. Animals are typically sampled in autumn, and the aetiological agents associated with respiratory disease in these cattle are likely to differ from those associated with neonatal calf pneumonia due to distinct seasonal differences in host and environmental factors. Co-infection with more than one respiratory pathogen was common on both farm types in the present study, consistent with findings of a recent Irish study [39] and supporting the multifactorial nature of respiratory disease in cattle.

Rotavirus and *Cryptosporidium parvum* were the pathogens most frequently identified in faecal samples taken from heifers with clinical signs of diarrhoea during the first visit period on both farm types. These findings are consistent with previous Irish and international studies, albeit at a lower detection rate [25,26,40,41]. Mixed infections were relatively uncommon (<6% of calves) among diarrhoeic calves on both farm types in this study when compared to similar studies [42]. Failure to detect other pathogens associated with calf diarrhoea and the large proportion of samples negative for all pathogens tested in this study could reflect the wide distribution, and relatively older age, of heifers in the sampled population (median age; 27 days, range 1–90 days). Regardless of the causative agent of neonatal calf diarrhoea, the clinical signs of infection are similar, with calves typically presenting with profuse diarrhoea, dehydration, and acidosis [43]. Cryptosporidiosis most commonly affects calves under 1 month old, and the period of risk of infection is greatest in calves between 9 and 12 days of age [44]. Similarly, rotaviral diarrhoea is most common in calves under 3 weeks old, with calves being at greatest risk of infection at 6 days old [45]. Additionally, because each calf was sampled on one occasion only, the intermittent shedding of enteric pathogens may have resulted in the under-diagnosis of the causative agents [46]. Pathogenic *E. coli* and coronavirus were not detected in any of the samples taken from study heifers. These findings are in agreement with those of an Irish study conducted on faecal samples from approximately 1800 calves with neonatal calf diarrhoea during 2018, where 1.4% and 0.6% were positive for *E. coli* and coronavirus, respectively [25]. Similarly low prevalence estimates of these pathogens have been reported in several European studies [40,47].

In approximately 60% of samples taken in the current study, no enteric pathogen was detected. This was considerably higher than in similar studies, which have reported pathogen detection failure rates of 4–28% [40,48,49,50]. Possible explanations for our findings are firstly, the relatively small sample size (*n* = 221) of faecal swabs tested; secondly, the use of a rapid immunoassay instead of a more sensitive diagnostic technique (such as qRt-PCR); and thirdly, the older median age of sampled animals. There was no association between farm type and the frequency of pathogens detected. This was not surprising, given that similarities in farm management and biosecurity practices during the neonatal period on both farm types [1], as discussed above.

Most commonly, source dairy farmers sent heifers to single-source rearing units; as a result, commingling of heifers from multiple origins was unlikely to occur on these farms. However, for 30% of SDFs, it was hypothesised that opportunities for commingling and/or direct contact with other cattle at the rearing unit would result in heifers being at increased risk of exposure to novel pathogens [51]. This was not found to be the case in this study, and the commingling of heifers at the rearing unit was not associated with abnormal health scores, with the exception of pyrexia (≥39.5 °C) during the first autumn visit period. These results are consistent with the findings of a study by Wiegand et al. [52], who found no association between the commingling of heifers from up to four sources with unknown health status and incidence of respiratory disease on a U.S. feedlot. Additionally, few associations were found between heifer health on source dairy farms and the number of farms sending heifers to the rearing unit.

The majority of SDFs in this study sent heifers to rearing units within the same county; therefore, travel distances between the source farm and rearing unit were relatively short (<100 km). As a result, contract-reared heifer calves were unlikely to experience many of the adverse health effects associated with long transport duration [53], a common feature of heifer rearing in other regions [54,55].

The health profiles of the heifers examined in this study suggest that these were generally well-managed herds. Results from less well-managed herds with more endemic disease could arguably have been different. Additionally, given the number of health parameters considered here, there was a potential for type 1 errors. This should be considered, along with the magnitude of the differences detected and the p-values reported when interpreting results. In order to provide a perspective on whether the differences observed are likely to be not just statistically, but also biologically significant and consistent with prior research, we have discussed our findings against the backdrop of the relevant international literature.

The implications of our findings for the contract heifer rearing as an enterprise are twofold. Firstly, we have shown that sending heifers off-site to a specialist rearing unit does not result in unfavourable heifer health outcomes. This may serve to assist the growth of the contract-rearing industry in Ireland. Secondly, for other cattle production systems that involve the commingling of animals from multiple sources with unknown disease status, such as the dairy calf and store, to beef systems, our findings may serve to reassure farmers, in principle, of the minimal disease risk associated with these practices, although further enterprise-specific research is warranted to confirm this hypothesis.

## 5. Conclusions

The main question addressed by this research was the difference between the farm of origin versus commercial rearing systems in the health of female dairy replacements. Contract-rearing did not impact health outcomes for replacement heifers when compared to heifers who spent the duration of the rearing period on their farm of origin. The number of source dairy farms at the rearing unit and commingling of heifers from multiple origins were not associated with an increased incidence of respiratory disease or diarrhoea among contract-reared heifers. The findings of this research are both topical and important for pastoral dairy industries worldwide.

## Figures and Tables

**Table 1:** Calf health scoring system, adapted from the University of Wisconsin Madison calf health scoring system.

Clinical Parameter	0	1	2	3
Nasal Discharge	Normal, serous discharge	Small amount of unilateral, cloudy discharge	Bilateral, cloudy or excessive mucus	Copious, bilateral mucopurulent nasal discharge
Ocular Discharge	Normal	Mild ocular discharge	Moderate bilateral ocular discharge	Heavy ocular discharge
Cough	No cough	Induce single cough	Induce repeated coughs or occasional spontaneous cough	Repeated spontaneous coughing
Rectal Temperature (°C)	37.8–38.3	38.4–38.8	38.9–39.4	≥39.5
Faecal consistency	Normal	Semi-formed, pasty	Loose, but stays on top of bedding	Watery, sifts through bedding
Navel	Normal	Slightly enlarged, not warm or painful	Slightly enlarged with slight pain or moisture	Enlarged with pain, heat or malodorous discharge
Joints	Normal	Slight swelling, not warm or painful	Swelling with pain or heat, slight lameness	Swelling with severe pain, heat and lameness

**Table 2:** Bacterial and viral agents that form part of the standard panel for investigation of bovine respiratory disease (BRD) outbreaks at the Department of Agriculture, Food and the Marine (DAFM) central veterinary research laboratory (CVRL).

Bacteriology (Culture)	Virology (RT-PCR)
*Histophilus somni*	Bovine herpesvirus 1 (BHV1)
*Mycoplasma bovis*	Bovine herpesvirus 4 (BHV4)
*Mannheimia haemolytica*	Bovine respiratory syncytial virus (BRSV)
*Pasteurella multocida*	Bovine parainfluenza virus-3 (PI3)
	Bovine coronavirus (BoCoV)

**Table 3:** Sensitivity and specificity of Zoetis Witness^®^ BOVID-5 faecal test kit compared to PCR and ELISA diagnostic techniques.

Pathogen	Sensitivity (%)	Specificity (%)	
*Cryptosporidium* spp.	96.8	98.2	vs. PCR
Coronavirus	96	95.3	
*E. coli* K99	96.8	97.9	
Rotavirus	96.4	95.9	
*Giardia lamblia*	96.5	97.3	vs. ELISA

**Table 4:** Mean (95% CI) within-herd prevalence (%) of abnormal health score outcomes (diarrhoea, BRD (bovine respiratory disease) and navel ill) in heifers, by herd size, (small *n* = 39, medium *n* = 37, large *n* =37) during the initial farm visit (spring 2018) when all heifers were scored on their farm of origin ^1^.

	Herd Size			
	Small (≤120 Cows)	Medium (121–200 Cows)	Large (≥201 Cows)	*p*-Value
Pyrexia (≥39.5 °C)	10.5 (6.76–13.35)	13.26 (9.88–16.65)	10.59 (7.11–14.07)	0.797
Pyrexia (≥40 °C)	1.41 (0.4–2.44)	2.45 (1.38–3.5)	2 (0.9–3.07)	0.409
Nasal ≥ 1	0.35 (0–2.2)	1.8 (0–3.68)	2.3 (0.4–4.1)	0.325
Eye ≥ 1	1.2 (0–2.8)	2.3 (0.6–4)	1.07 (0–2.8)	0.514
Cough ≥ 1	1.5 (0–3.3) ^a^	4.7 (2.8–6.5) ^c^	2 (2.2–5.0) ^b^	0.039
Faecal ≥ 1	3.7 (1.2–6.2)	6 (3.4–8.6)	5 (2.39–7.5)	0.450
Navel ill	1.1 (0–2.3)	1.5 (0.2–2.7)	1.3 (0.6–2.6)	0.909
BRD	4.1 (0–8.86)	5.3 (0.4–10.2)	4.4 (0–9.3)	0.938
Diarrhoea	3.2 (1.2–5.3)	4.8 (2.7–7)	3.6 (1.5–5.8)	0.566
At least 1 abnormal score	16.5 (9.2–24)	20.7 (13.1–28.2)	18.6 (11–26)	0.741
Multiple abnormal scores	6.5 (3.6–9.4) ^a^	11.3 (8.3–14.2) ^c^	8.4 (5.4–11.3) ^b^	0.077

^a^ < ^b^ < ^c^, ^1^ Data are presented with 95% confidence intervals in parenthesis.

**Table 5:** Distribution frequencies of abnormal health score outcomes (% of calves) on farms where heifers were reared offsite at specialist contract-rearing farms (SDF, source dairy farm) and farms where heifers were reared onsite (CF, control dairy farm) over four sampling periods (*n* = 5532).

Farm Type	Sampling Period	Temp Score = 1 (≥39.5) (%)	*p*-Value	Faecal Score ≥1 (%)	*p*-Value	Nasal Score ≥1 (%)	*p*-Value	Eye Score ≥1 (%)	*p*-Value	Cough Score ≥1 (%)	*p*-Value	Navel Score ≥1 (%)	*p*-Value	Joint Score ≥1 (%)	*p*-Value
CF	S1 ^1+^	11.8	0.782	4.7	0.935	0.5	0.001 *	1.1	0.042 *	1.5	0.225	9.8	0.013 *	0	0.03 *
SDF		11. 6		4.7		3.5		1.9		2		7.7		0.3	
CF	A1 ^2^	14.9	0.001 *	0.2	0.003 *	2.2	0.001 *	0	NA	0.9	0.796				
SDF		8.1		0.9		3.8		0		1					
CF	S2 ^3^	4.9	0.001 *	0.6	0.48	0.6	0.48	0	0.296	0.2	0.566				
SDF		2.7		0.8		0.8		0.1		0.1					
CF	A2 ^4^	2.3	0.411	0	1	0.2	1	0	0.123	0	1				
SDF		3.1		0		0.2		0.1		0					

^1^ S1; spring 1, heifers < 6 months old, ^2^ A1; autumn 1, heifers approximately 8 months old, ^3^ S2; spring 2, heifers approximately 12 months old, ^4^ A2; autumn 2, heifers approximately 20 months old. * Significant at *p* < 0.05 ^+^. During the initial farm visit, heifers were scored on their source dairy farm prior to movement to the rearing unit. NA; not applicable, no coronavirus detected.

**Table 6:** Overall mean within-herd prevalence (%) of abnormal health score outcomes, diarrhoea, BRD (bovine respiratory disease) and navel ill heifers over four sampling periods.

		Sampling Period		
	S1 ^1^	A1 ^2^	S2 ^3^	A2 ^4^
Pyrexia (≥39.5 °C)	11.29 (±10.6)	10.9 (±13.7)	3.8 (±5.4)	2.5 (±3.2)
Pyrexia (≥40 °C)	1.9 (±3.3)	1.6 (±3.14)	0.38 (±1.65)	0
Nasal ≥ 1	1.4 (±5.8)	3.5 (±6.3)	0.75 (±1.6)	0.12 (±0.6)
Eye ≥ 1	1.5 (±5.2)	0	0.19 (±0.15)	0.05 (±0.43)
Cough ≥ 1	3.4 (±5.7)	0.9 (±2)	0.16 (±0.91)	0.04 (±0.25)
Faecal ≥ 1	4.9 (±7.9)	0.5 (±2.28)	0.6 (±1.75)	0
BRD	4.6 (±14.9)	3.14 (±10.5)	1.23 (±9.5)	0
Navel ill	1.3 (±3.8)			
Diarrhoea	3.9 (±6.62)	0.6 (±2.27)	0.6 (±1.7)	0
Multiple abnormal scores	8.7 (±9.3)	0.87 (±2.5)	0.08 (±0.5)	0
At least 1 abnormal score	18.6 (±23)	14.3 (±14)	5.2 (±5.6)	0.2 (±0.7)

^1^ S1; spring 1, heifers < 6 months old, ^2^ A1; autumn 1, heifers approximately 8 months old, ^3^ S2; spring 2, heifers approximately 12 months old, ^4^ A2; autumn 2, heifers approximately 20 months old. Data are presented with the standard deviation (SD) in parenthesis.

**Table 7:** Association between farm type (SDF, source dairy farm; CF, control farm) and mean within-herd prevalence (%) of abnormal health score outcomes, diarrhoea, BRD (bovine respiratory disease) and navel ill in heifers over four sampling periods (S1; spring 1, heifers < 6 months old, A1; autumn 1, heifers approximately 8 months old, S2; spring 2, heifers approximately 12 months old, A2; autumn 2, heifers approximately 20 months old).

	Farm Type											
	S1			A1			S2			A2		
	SDF	CF	*p*-Value	SDF	CF	*p*-Value	SDF	CF	*p*-Value	SDF	CF	*p*-Value
Pyrexia (≥39.5 °C)	10.9 (8.2–13.6)	11.8 (8.8–14.9)	0.648	8.36 (4.9–11.8)	13.9 (10.1–17.6)	0.035 *	2.9 (1.6–4.2)	4.8 (3.3–6.3)	0.062	3.1 (1.7–4.6)	2 (0.58–3.4)	0.257
Pyrexia (≥40 °C)	1.8 (0.97–2.6)	2.1 (1.2–3)	0.632	1.3 (0.5–2.1)	2 (1.2–2.9)	0.282	0.3 (0–0.72)	0.5 (0.24–0.9)	0.556			
Nasal ≥ 1	2.4 (0.99–3.9)	0.27 (0–1.8)	0.048 *	4 (2.4–5.7)	2.7 (1–4.5)	0.258	0.785 (0.4–1.2)	0.7 (0.6–1.2)	0.792	0.1 (0–0.25)	0.14 (0–0.3)	0.785
Eye ≥ 1	1.5 (0.2–2.8)	1.6 (0.1–3)	0.932	0	0		0	0				
Cough ≥ 1	4.8 (3.4–6.2)	1.6 (0.9–3.15)	0.003 *	0.9 (3.8–1.4)	1 (0.4–1.5)	0.881	0.16 (0–0.4)	0.16 (0–0.42)	0.603			
Faecal ≥ 1	4.9 (2.9–6.9)	4.8 (2.6–7)	0.943	0.9 (0.3–1.1)	0.3 (0–0.9)	0.157	0.67 (0.22–1.1)	0.55 (0.6–1)	0.716			
BRD	6.1 (2.3–9.8)	2.8 (0–7)	0.24	3.72 (1.1–6.4)	2.5 (0–5.4)	0.526	1.9 (0–4.3)	0.4 (0–3)	0.413			
Navel ill	1.4 (0.44–2.4)	1.15 (0.08–2.2)	0.72									
Diarrhoea	3.7 (2–5.4)	4.1 (2.3–6)	0.736	0.9 (0.3–1.4)	0.3 (0–8.78)	0.159	0.67 (0.23–1)	0.5 (0.2–1)	0.621			
Multiple abnormal scores	9 (6.6–11.3)	8.3 (5.7–10.9)	0.72	0.8 (0.2–1.4)	0.96 (0.27–1.7)	0.739	0.07 (0–0.19)	0.1 (0–0.238)	0.726			
At least 1 abnormal score	18.4 (12.6–24.2)	18.6 (12.3–25.2)	0.938	12.4 (8.9–16)	16.5 (12.7–20.4)	0.122	4.5 (3.1–5.9)	6 (4.5–7.6)	0.146	1 (0–0.28)	0.26 (0.06–0.45)	0.246

Data are presented with 95% confidence intervals in parenthesis. * Significant at *p* < 0.05.

**Table 8:** Distribution of respiratory pathogens (% of calves) in nasal samples taken from heifer calves under 6 months old with clinical signs of respiratory disease (SDF, source dairy farm; CF, control dairy farm; *n*, number of samples tested).

Respiratory Pathogen	SDF (%)	CF (%)	*p*-Value
*H. somni*	2 (*n =* 51)	11 (*n =* 27)	0.117
*M. bovis*	0 (*n =* 51)	22 (*n =* 27)	0.001
*M. haemolytica*	42 (*n =* 48)	27 (*n =* 26)	0.028
*P. multocida*	76 (*n =* 46)	65 (*n =* 26)	0.330
BHV-1	0 (*n =* 51)	0 (*n =* 27)	
BHV-4	0 (*n =* 51)	0 (*n =* 27)	
BoCoV	0 (*n =* 51)	4 (*n =* 27)	0.346
BRSV	0 (*n =* 48)	4 (*n =* 25)	0.342
PI3	2 (*n =* 51)	8 (*n =* 23)	0.262

BHV-1, Bovine herpesvirus-1; BHV-4, Bovine herpesvirus 4; BoCoV, Bovine coronavirus; BRSV, Bovine respiratory syncytial virus; PI3, Parainfluenza virus 3.

**Table 9:** Distribution of nasal swab results by number of pathogens detected per sample on source (SDF) and control (CF) dairy farms (*n*, number of samples tested).

	SDF (%)	CF (%)	*p*-Value
Positive for at least 1 pathogen	77 (*n* = 51)	85 (*n* = 27)	0.365
Positive for ≥2 pathogens	39 (*n* = 39)	52 (*n* = 23)	0.293

**Table 10:** Distribution of pathogens (% of calves) detected in samples taken from heifer calves under 6 months old with clinical signs of diarrhoea on source (SDF) and control (CF) dairy farms.

Pathogen	SDF (%) (*n* = 157)	CF (%) (*n* = 64)	*p*-Value
*C. parvum*	17	17	0.91
Rotavirus	30	28	0.789
Coronavirus	0	0	
*E. coli* K99	0	0	
*Giardia lamblia*	0	0	
*Salmonella* Dublin	0	0	

*C. parvum*: *Cryptosporidium parvum*.

**Table 11:** Distribution of faecal swab results by the number of pathogens detected per sample on source (SDF) and control (CF) dairy farms.

	SDF (%) (*n* = 157)	CF (%) (*n* = 64)	*p*-Value
Positive for at least one pathogen	41	42	0.845
Positive for *C. parvum* and rotavirus	6	3	0.517

**Table 12:** Mean (95% CI) within-herd prevalence (%) of abnormal health score outcomes [diarrhoea, BRD (bovine respiratory disease) and navel ill] in contract (CR, contract-reared) heifers over three sampling periods by the number of source dairy farmers sending heifers to the rearing unit.

	Number of Farms at Contract–Rearing Unit								
	A1 ^1^			S2 ^2^			A2 ^3^		
	1	>1	*p*-Value	1	>1	*p*-Value	1	>1	*p*-Value
Pyrexia (≥39.5 °C)	7.8 (4.7–11)	9.1 (5.3–12.9)	0.611	2.7 (1.5–3.9)	3.3 (1.78–4.81)	0.534	3.73 (1.3–6.2)	2.6 (0.41–4.8)	0.486
Pyrexia (≥40 °C)	1 (0.23–1.8)	1.8 (0.8–2.7)	0.245	0.24 (0–0.53)	0.4 (0.024–0.77)	0.008 *			
Nasal ≥ 1	4.2 (2–6.3)	4 (1.4–6.4)	0.871	0.94 (0.42–1.5)	0.56 (0–1.2)	0.357	0.18 (0.05–0.31)	0	0.095
Cough ≥ 1	0.54 (0–1.24)	1.4 (0.57–2.26)	0.116	0.27 (0–0.62)	0	0.326	0.13 (0.02–0.238)	0	0.152
Faecal ≥ 1	0.325 (0–1.3)	1.6 (0.51–2.77)	0.08	0.7 (0.13–1.3)	0.6 (0–1.3)	0.758	0	0	
BRD	2.1 (0–6.4)	6 (0.88–11.2)	0.246	0.44 (0–4.64)	4.17 (0–9.38)	0.27	0	0	
Diarrhoea	0.33 (0–1.26)	1.6 (0.5–2.8)	0.081	0.72 (0.13–1.3)	0.6 (0–1.3)	0.492	0	0	
Multiple abnormal scores	0.67 (0.7–1.3)	1 (0.29–1.7)	0.476				0	0	
At least 1 abnormal score	11.2 (7.9–14.5)	14.2 (10.3–18.2)	0.239	4.6 (3.1–6.1)	4.4 (2.6–6.2)	0.913	0.16 (0.04–0.29)	0	0.112

Data are presented with 95% confidence intervals in parenthesis. * Significant at *p* < 0.05. ^1^ A1; autumn 1 sampling period, heifers approximately 8 months old, ^2^ S2; spring 2 sampling period, heifers approximately 12 months old, ^3^ A2; autumn 2 sampling period, heifers approximately 20 months old.

**Table 13:** Association between the commingling of heifers at the rearing unit and mean within-herd prevalence (%) of abnormal health score outcomes, diarrhoea, BRD (bovine respiratory disease) and navel ill in contract (contract-reared; CR) heifers over three sampling periods.

	Commingling of Heifers at Contract-Rearing Unit								
	A1 ^1^			S2 ^2^			A2 ^3^		
	Yes	No	*p*-Value	Yes	No	*p*-Value	Yes	No	*p*-Value
Pyrexia (≥39.5 °C)	9 (3.6–14.6)	8.18 (5.5–10.9)	0.769	2.75 (0.5–5)	2.97 (1.9–4)	0.86	3.5 (1.5–5.4)	2.3 (0–5.3)	0.518
Pyrexia (≥40 °C)	2.8 (1.5–4.1)	0.98 (0.32–1.6)	0.016 *	0.18 (0–0.73)	0.324 (0.07–0.58)	0.642	0	0	
Nasal ≥ 1	5.5 (1.9–9.1)	3.7 (1.9–5.5)	0.374	0.7 (0–1.6)	0.8 (0.36–1.25)	0.851	0	0.132	0.323
Cough ≥ 1	1.5 (0.3–2.72)	0.75 (0.14–1.36)	0.285	0	0.2 (0–0.5)	0.56	0	0.09 (0–0.19)	0.396
Faecal ≥ 1	2.25 (0.6–3.85)	0.53 (0–1.3)	0.067	0	0.83 (0.32–1.3)	0.152	0	0	
BRD	3.12 (0–10.7)	3.9 (0–7.6)	0.859	0	2.3 (0–6)	0.589	0	0	
Diarrhoea	2.25 (0.6–3.8)	0.52 (0–1.3)	0.065	0	0.83 (0.33–1.3)	0.152	0	0	
Multiple abnormal scores	1.5 (0.5–2.5)	0.63 (0.13–1.13)	0.131	0	0.08 (0–0.23)	0.63	0	0	
At least 1 abnormal score	15.2 (9.4–20.9)	11.8 (8.9–14.6)	0.291	3.5 (0.91–6.1)	4.8 (3.5–6)	0.385	0	0.12 (0.009–0.231)	0.346

Data are presented with 95% confidence intervals in parenthesis. * Significant at *p* < 0.05. ^1^ A1; autumn 1 sampling period, heifers approximately 8 months old, ^2^ S2; spring 2 sampling period, heifers approximately 12 months old, ^3^ A2; autumn 2 sampling period, heifers approximately 20 months old.

## Data Availability

Data are available from the corresponding author, upon reasonable request.

## References

[1] McCarthy M.C., O’Grady L., McAloon C.G., Mee J.F. (2021). A survey of biosecurity and health management practices on Irish dairy farms engaged in contract-rearing. J. Dairy Sci..

[2] Mee J.F., Geraghty T., O’Neill R., More S.J. (2012). Bioexclusion of diseases from dairy and beef farms: Risks of introducing infectious agents and risk reduction strategies. Vet. J..

[3] Taylor J.D., Fulton R.W., Lehenbauer T.W., Step D.L., Confer A.W. (2010). The epidemiology of bovine respiratory disease: What is the evidence for predisposing factors?. Can. Vet. J..

[4] Johnson K.F., Chancellor N., Wathes D.C. (2021). A Cohort Study Risk Factor Analysis for Endemic Disease in Pre-Weaned Dairy Heifer Calves. Animals.

[5] Carroll J.A., Forsberg N.E. (2007). Influence of Stress and Nutrition on Cattle Immunity. Vet. Clin. N. Am. Food Anim. Pract..

[6] Weaver D.M., Tyler J.W., VanMetre D.C., Hostetler D.E., Barrington G.M. (2000). Passive transfer of colostral immunoglobulins in calves. J. Vet. Intern. Med..

[7] Stanton A., Kelton D., LeBlanc S., Wormuth J., Leslie K. (2012). The effect of respiratory disease and a preventative antibiotic treatment on growth, survival, age at first calving, and milk production of dairy heifers. J. Dairy Sci..

[8] Stanton A., Kelton D., LeBlanc S., Millman S., Wormuth J., Dingwell R., Leslie K.E. (2010). The effect of treatment with long-acting antibiotic at postweaning movement on respiratory disease and on growth in commercial dairy calves. J. Dairy Sci..

[9] Bach A. (2011). Associations between several aspects of heifer development and dairy cow survivability to second lactation. J. Dairy Sci..

[10] Warnick L., Erb H., White M. (1994). The association of calfhood morbidity with first-lactation calving age and dystocia in New York Holstein herds. Kenya Vet..

[11] Earley B., Arguello A., O’Riordan E., Crosson P., Cappelleri A., McGee M. (2019). Antimicrobial drug usage from birth to 180 days of age in Irish dairy calves and in suckler beef calves. J. Appl. Anim. Res..

[12] Todd C.G., McGee M., Tiernan K., Crosson P., O’Riordan E., McClure J., Lorenz I., Earley B. (2018). An observational study on passive immunity in Irish suckler beef and dairy calves: Tests for failure of passive transfer of immunity and associations with health and performance. Prev. Vet. Med..

[13] McGuirk S.M., Peek S.F. (2014). Timely diagnosis of dairy calf respiratory disease using a standardized scoring system. Anim. Health Res. Rev..

[14] O’Neill R., Mooney J., Connaghan E., Furphy C., Graham D. (2014). Patterns of detection of respiratory viruses in nasal swabs from calves in Ireland: A retrospective study. Vet. Rec..

[15] Fayer R., Ungar B. (1986). *Cryptosporidium* spp. and cryptosporidiosis. Microbiol. Rev..

[16] Zoetis (2016). Veterinary Products Compendium of Switzerland: Institute of Veterinary Pharmacology and Toxicology. https://www.vetpharm.uzh.ch/tpp/00000000/V0137-XX.HTM.

[17] Mahendran S.A., Booth R., Beekhuis L., Manning A., Blackmore T., Vanhoudt A., Bell N. (2017). Assessing the effects of weekly preweaning health scores on dairy calf mortality and productivity parameters: Cohort study. Vet. Rec..

[18] McGuirk S.M. (2008). Disease management of dairy calves and heifers. Vet. Clin. N. Am. Food Anim. Pract..

[19] Cramer M.C., Proudfoot K.L., Ollivett T.L. (2019). Short communication: Behavioral attitude scores associated with bovine respiratory disease identified using calf lung ultrasound and clinical respiratory scoring. J. Dairy Sci..

[20] Sinnott A.M., Kennedy E., Bokkers E.A. (2021). The effects of manual and automated milk feeding methods on group-housed calf health, behaviour, growth and labour. Livest. Sci..

[21] Conneely M., Berry D., Murphy J.P., Lorenz I., Doherty M.L., Kennedy E. (2014). Effect of feeding colostrum at different volumes and subsequent number of transition milk feeds on the serum immunoglobulin G concentration and health status of dairy calves. J. Dairy Sci..

[22] Raboisson D., Delor F., Cahuzac E., Gendre C., Sans P., Allaire G. (2013). Perinatal, neonatal, and rearing period mortality of dairy calves and replacement heifers in France. J. Dairy Sci..

[23] Reiten M., Rousing T., Thomsen P., Otten N., Forkman B., Houe H., Sørensen J.T., Kirchner M.T. (2018). Mortality, diarrhea and respiratory disease in Danish dairy heifer calves: Effect of production system and season. Prev. Vet. Med..

[24] Cuttance E., Mason W., McDermott J., Laven R., McDougall S., Phyn C. (2017). Calf and replacement heifer mortality from birth until weaning in pasture-based dairy herds in New Zealand. J. Dairy Sci..

[25] DAFM (2020). All-Island Animal Disease Surveillance Report 2019.

[26] Al Mawly J., Grinberg A., Prattley D., Moffat J., Marshall J., French N. (2015). Risk factors for neonatal calf diarrhoea and enteropathogen shedding in New Zealand dairy farms. Vet. J..

[27] Lorenz I., Mee J.F., Earley B., More S.J. (2011). Calf health from birth to weaning. I. General aspects of disease prevention. Ir. Vet. J..

[28] Svensson C., Lundborg K., Emanuelson U., Olsson S.-O. (2003). Morbidity in Swedish dairy calves from birth to 90 days of age and individual calf-level risk factors for infectious diseases. Prev. Vet. Med..

[29] Sivula N.J., Ames T.R., Marsh W.E., Werdin R.E. (1996). Descriptive epidemiology of morbidity and mortality in Minnesota dairy heifer calves. Prev. Vet. Med..

[30] Urie N.J., Lombard J.E., Shivley C.B., Kopral C.A., Adams A.E., Earleywine T.J., Olson J.D., Garry F.B. (2018). Preweaned heifer management on US dairy operations: Part V. Factors associated with morbidity and mortality in preweaned dairy heifer calves. J. Dairy Sci..

[31] Walker W., Epperson W., Wittum T.E., Lord L.K., Rajala-Schultz P.J., Lakritz J. (2012). Characteristics of dairy calf ranches: Morbidity, mortality, antibiotic use practices, and biosecurity and biocontainment practices. J. Dairy Sci..

[32] Dachrodt L., Arndt H., Bartel A., Kellermann L.M., Tautenhahn A., Volkmann M., Birnstiel I., Do Duc P., Hentzsch A., Jensen K.C. (2021). Prevalence of disorders in preweaned dairy calves from 731 dairies in Germany: A cross-sectional study. J. Dairy Sci..

[33] Love W.J., Lehenbauer T.W., Van Eenennaam A.L., Drake C.M., Kass P.H., Farver T.B., Aly S.S. (2016). Sensitivity and specificity of on-farm scoring systems and nasal culture to detect bovine respiratory disease complex in preweaned dairy calves. J. Vet. Diagn. Investig..

[34] Fischer A., Song Y., He Z., Haines D., Guan L., Steele M. (2018). Effect of delaying colostrum feeding on passive transfer and intestinal bacterial colonization in neonatal male Holstein calves. J. Dairy Sci..

[35] Van der Fels-Klerx H.J., Horst H.S., Dijkhuizen A.A. (2000). Risk factors for bovine respiratory disease in dairy youngstock in The Netherlands: The perception of experts. Livest. Prod. Sci..

[36] Jensen M.B., Herskin M.S., Rørvang M.V. (2019). Secluded maternity areas for parturient dairy cows offer protection from herd members. J. Dairy Sci..

[37] Svensson C., Hultgren J., Oltenacu P.A. (2006). Morbidity in 3–7-month-old dairy calves in south-western Sweden, and risk factors for diarrhoea and respiratory disease. Prev. Vet. Med..

[38] Barry J., Bokkers E.A.M., Berry D.P., de Boer I.J.M., McClure J., Kennedy E. (2019). Associations between colostrum management, passive immunity, calf-related hygiene practices, and rates of mortality in preweaning dairy calves. J. Dairy Sci..

[39] Murray G.M., More S.J., Sammin D., Casey M.J., McElroy M.C., O’Neill R.G., Byrne W.J., Earley B., Clegg T.A., Ball H. (2017). Pathogens, patterns of pneumonia, and epidemiologic risk factors associated with respiratory disease in recently weaned cattle in Ireland. J. Vet. Diagn. Investig..

[40] Lanz Uhde F., Kaufmann T., Sager H., Albini S., Zanoni R., Schelling E., MedVet M., Habil F.V.H. (2008). Prevalence of four enteropathogens in the faeces of young diarrhoeic dairy calves in Switzerland. Vet. Rec..

[41] Izzo M., Kirkland P., Mohler V., Perkins N., Gunn A., House J. (2011). Prevalence of major enteric pathogens in Australian dairy calves with diarrhoea. Aust. Vet. J..

[42] Garcıa A., Ruiz-Santa-Quiteria J., Orden J., Cid D., Sanz R., Gómez-Bautista M., de la Fuente R. (2000). Rotavirus and concurrent infections with other enteropathogens in neonatal diarrheic dairy calves in Spain. Comp. Immunol. Microbiol. Infect. Dis..

[43] Bazeley K. (2003). Investigation of diarrhoea in the neonatal calf. Practice.

[44] Castro-Hermida J.A., González-Losada Y.A., Mezo-Menéndez M., Ares-Mazás E. (2002). A study of cryptosporidiosis in a cohort of neonatal calves. Vet. Parasitol..

[45] Foster D.M., Smith G.W. (2009). Pathophysiology of Diarrhea in Calves. Vet. Clin. N. Am. Food Anim. Pract..

[46] Gulliksen S.M., Jor E., Lie K.I., Hamnes I.S., Løken T., Åkerstedt J., Østerås O. (2009). Enteropathogens and risk factors for diarrhea in Norwegian dairy calves. J. Dairy Sci..

[47] Bartels C.J., Holzhauer M., Jorritsma R., Swart W.A., Lam T.J. (2010). Prevalence, prediction and risk factors of enteropathogens in normal and non-normal faeces of young Dutch dairy calves. Prev. Vet. Med..

[48] De la Fuente R., García A., Ruiz-Santa-Quiteria J.A., Luzón M., Cid D., García S., Orden J.A., Gómez-Bautista M. (1998). Proportional morbidity rates of enteropathogens among diarrheic dairy calves in central Spain. Prev. Vet. Med..

[49] Abuelo A., Havrlant P., Wood N., Hernandez-Jover M. (2019). An investigation of dairy calf management practices, colostrum quality, failure of transfer of passive immunity, and occurrence of enteropathogens among Australian dairy farms. J. Dairy Sci..

[50] Al Mawly J., Grinberg A., Prattley D., Moffat J., French N. (2015). Prevalence of endemic enteropathogens of calves in New Zealand dairy farms. N. Z. Vet. J..

[51] Step D., Krehbiel C., DePra H., Cranston J., Fulton R., Kirkpatrick J., Gill D.R., Payton M.E., Montelongo M.A., Confer A.W. (2008). Effects of commingling beef calves from different sources and weaning protocols during a forty-two-day receiving period on performance and bovine respiratory disease. J. Anim. Sci..

[52] Wiegand J.B., Cooke R.F., Brandão A.P., Schubach K.M., Colombo E.A., Daigle C.L., Duff G.C., Gouvêa V.N. (2020). Impacts of commingling cattle from different sources on their physiological, health, and performance responses during feedlot receiving. Transl. Anim. Sci..

[53] Masmeijer C., Devriendt B., Rogge T., van Leenen K., De Cremer L., Van Ranst B., Deprez P., Cox E., Pardon B. (2019). Randomized field trial on the effects of body weight and short transport on stress and immune variables in 2- to 4-week-old dairy calves. J. Vet. Intern. Med..

[54] Celestino M.L., Menta P.R., Fernandes L., Poit D., Neves R.C., Ballou M.A., Caixeta L.S., Machado V.S. (2021). Short communication: Associations of serum biomarkers of stress and inflammation measured at arrival with health, mortality, and growth of calves transported within the first 4 days of life. J. Dairy Sci..

[55] USDA-NAHMS (2007). Reference of Dairy Cattle Health and Management Practices in the United States.

